# Enhanced sensitivity of neutralizing antibody detection for different AAV serotypes using HeLa cells with overexpressed AAVR

**DOI:** 10.3389/fphar.2023.1188290

**Published:** 2023-04-27

**Authors:** Zhaoyue Zheng, Jingya Ye, Mi Leng, Chunmei Gan, Na Tang, Wei Li, C. Alexander Valencia, Biao Dong, Hoi Yee Chow

**Affiliations:** ^1^ National Clinical Research Center for Geriatrics and State Key Laboratory of Biotherapy, West China Hospital, Sichuan University, Chengdu, China; ^2^ Sichuan Real and Best Biotech Co., Ltd., Chengdu, China; ^3^ Department of Dermatovenereology, Rare Disease Center, West China Hospital, Sichuan University, Chengdu, China

**Keywords:** gene therapy, adeno-associated virus, neutralizing antibody, AAVR, stable cell line

## Abstract

A cell-based transduction inhibition assay (TI) is widely used in clinical trials to detect neutralizing antibody (NAb) titers against recombinant adeno-associated virus (rAAV), one of the most important criteria to exclude patients in gene therapy. Different cell lines are used in cell-based TI because the rAAV transduction efficiencies vary largely among serotypes. A cell line suitable for TI for most serotypes is highly desirable, especially for those with very low transduction efficiencies *in vitro* such as rAAV8 and rAAV9. Herein, we report an AAVR-HeLa, a stable cell line with overexpressed AAVR, a newly identified receptor for rAAVs, was established for cell-based TIs. The AAVR expression level in AAVR-HeLa cells was approximately 10-fold higher than in HeLa cells, and was stably transfected after twenty three passages. For all AAV serotypes (AAV1-10), except for AAV4, the transduction efficiencies increased significantly in AAVR-HeLa cells. It was demonstrated that the AAVR enhancement of transduction efficiency was only for rAAV and not for lentiviral and adenoviral vectors. According to the minimal multiplicity of infection (MOIs) for the assay, the NAb detection sensitivity increased at least 10 and 20 fold for AAV8 and AAV9, respectively. The seroprevalence of NAbs were investigated at the 1:30 level as a cutoff value using AAVR-HeLa cells. It was shown that the seropositive rate for AAV2 was 87% in serum samples from 99 adults, followed by lower seropositive rates for AAV5 (7%), AAV8 (7%) and AAV9 (1%). Venn diagram analysis showed the presence of cross-reactivity of NAbs to two or three serotypes in 13 samples (13.1%). However, no patient was found to possess NAbs for all the four serotypes. These results demonstrated that the AAVR-HeLa cell line may be utilized to detect the NAbs through cell-based TI assays for most of AAV serotypes.

## Introduction

AAV is one of the most promising viral vectors for gene therapy due to its excellent safety, long-term expression, and high infectivity to a broad range of cell types (McCarty et al., 2004; Li et al., 2011; Gaj et al., 2016). Nearly 250 rAAV-mediated therapies have been used in clinical trials, and six approved for commercial use (Bryant et al., 2013; Smalley, 2017; Dangouloff and Servais, 2019; Kirschner et al., 2020; Keam, 2022; Ozelo et al., 2022; Heo, 2023). More than 100 AAV serotypes have been identified from human and non-human tissues (Wu et al., 2006) and some AAV serotypes found to infect humans widely (Verdera et al., 2020). To further increase the transduction efficiency in a specific tissue or cell type, many new serotypes were selected using various strategies including capsid evolution (Ran et al., 2020). It has been reported that the healthy population exposure to AAV2 is 59% and to AAV1 is 50.5% (Boutin et al., 2010). Pre-existing NAbs to AAV in humans can bind to rAAV vectors, and the NAb titer is as low as 1:5 may fully inhibit their infection (Wang et al., 2011; Guo et al., 2019), leading to the therapy failure. To escape the immune response, rAAV shielding such as nanoparticle coating was used widely (Trigueros et al., 2019). Optimization of the manufacturing process was also used for this purpose (Zheng et al., 2021).

Three major assays have been used to detect pre-existing NAbs to the AAV capsid (Falese et al., 2017), namely, an ELISA-based assay, an *in vivo* TI assay and a cell-based TI assay. The ELISA-based assay can be used to estimate the total binding antibodies (TAb) (Falese et al., 2017; Day et al., 2021), and has higher sensitivity than a cell-based TI assay. However, this method may cause a high TAb prevalence, which may lead to overestimate the immune effect of gene therapy due to their non-linear relationship in blocking activity at low titers (Masat et al., 2013). The *in vivo* TI assay is suitable for AAV serotypes that have low infection efficiency *in vitro*, but this method is difficult to standardize, more time-consuming and costly (Mendell et al., 2022). The cell-based TI assay is most widely used for NAb detection. It is easy to set up, and can detect both the antibody and other non-antibody neutralizing factors (Rincon et al., 2016). Currently, many clinical trials prefer the use of a cell-based TI assay to screen for NAb negative patients (Miesbach et al., 2018; Eggers et al., 2022; Mendell et al., 2022). However, the sensitivity of this assay is related to the infection efficiency of different rAAV serotypes and the reporter genes carried by rAAVs. The low efficiency of viral infections requires an increased MOI for detection, which leads to the reduction of the assay sensitivity (Masat et al., 2013; Mendell et al., 2022). To address this issue, several cell lines were selected according to transduction efficiencies for different AAV serotypes, namely, HEK293 for rAAV2 and rAAV8 (Kay et al., 2000; Meliani et al., 2015), COS-7 for rAAV3 (Daniel et al., 2022), GM16095 for rAAV2 (Guo et al., 2019), and HeLa for most serotypes. The modified cell line 2V6.11 was established for NAb detection by the overexpression of the adenovirus E4 ORF in HEK293 cells, which improves the transduction of rAAV6, rAAV8, and rAAV9 (Mohammadi et al., 2004; Meliani et al., 2015). Generally, the more efficient the rAAV infection, the higher the sensitivity of NAb detection.

AAVR is a newly identified universal receptor for AAV transduction and it mediates AAV transport from the plasma membrane to trans-Golgi (Pillay et al., 2016). Overexpression of AAVR was shown to increase the rAAV2 infection efficiency up to approximately 3-fold (Pillay et al., 2016). Similarly, rAAV2 transduction was enhanced by AAVR overexpression in the cell basolateral membrane (Hamilton et al., 2019). By co-transfection of AAVR, rAAV2 delivery efficiency in the neural retrograde pathway was highly increased (Sano et al., 2020), and rAAV8-based gene editing efficiency was increased up to 15-fold (McDougald et al., 2019; Yin et al., 2022). In sum, AAVR overexpression may be an important approach to augment AAV infection (Meyer and Chapman, 2022).

To leverage the capacity of AAVR to increase transduction efficiency, a stable HeLa cell line, AAVR-HeLa, was constructed to overexpress AAVR for NAb detection. By using AAVR-HeLa cells, a lower amount of viral vectors could be used for the NAb assay and the sensitivity of NAb detection could be increased, which was the aim of this study. Herein, we reported the NAb detection results in Chinese adults using the newly established AAVR-HeLa cells.

## Materials and methods

### Cell culture

HEK293 (CRL-1573), HEK293 T (CRL-3216), HeLa (CCL-2) cells were purchased from ATCC. All the cells were cultured in Dulbecco’s modified Eagle’s medium (Invitrogen, Carlsbad, CA) with 10% fetal bovine serum (Gibco, Gaithersburg, MD), supplied with 100 µg of penicillin/mL and 100 U of streptomycin/mL. The cells were maintained at 37°C incubator supplied with 5% CO_2_. The cells were prepared for subculture for every 3 days with the seeding density at 1 × 10^5^ cells/mL.

### Plasmid construction

To generate AAVR overexpression plasmid, AAVR cDNA sequence (NCBI code NM_024874.5) was synthesized and cloned into PLVX-Puro plasmid (Addgene#6108) via restriction sites BamHI and XbaI (NEB, Ipswich, MA). The plasmid was named PLVX-Puro-AAVR. The plasmids for rAAV packaging were pssAAV-CB-Luciferase, pdsAAV-CB-EGFP, and Ad helper plasmid (pAd) as previously described (Cao et al., 2002) (Wang et al., 2003). AAV helper plasmids containing the capsid sequences, pRC1-10, were synthesized by General Biology (Anhui, China).

### Establishment of a stable HeLa cell line

A Lentiviral vector was used to establish the stable cell line with AAVR overexpression. To generate the lentiviral vector, the plasmid PLVX-Puro-AAVR with two helper plasmids psPAX2 (Addgene#12260) and pMD2. G (Addgene#12259) were co-transfected into 293 T cells by Lipofectamine 2000 (Thermo Fisher Scientific, Waltham, MA) at a ratio of 4:2:1 according to the manufacturer’s protocol. The supernatants were harvested 48 h after transfection and centrifuged at 10,000 g to discard cell debris.

To generate the stable cell line with AAVR overexpression, HeLa cells were seeded onto a 100 mm dish and infected with the lentiviral vector with the MOI of 5. Forty-eight hours after infection, cells were passaged and selected with 1 μg/mL puromycin (Sangon Biotec, Shanghai, China) for at least three generations. Western blot confirmation of overexpression of AAVR in the stable cells, AAVR-HeLa, was performed followed by single colonies selections from the pool. Briefly, cells were seeded onto a 96-well plate at a density of 0.5 cell per well, supplied with 1 μg/mL puromycin in the growth media and maintained at 37°C with 5% CO_2_. The plate was incubated for 10–15 days, and the single cell colonies were selected for further passaging and confirmation by Western blotting. To test the stability of the selected AAVR-HeLa cell line, cells were passaged every 3 days for 23 generations. Cells from the odd number generations were used to evaluate the change of AAVR expression levels by Western blotting.

### rAAV production

A triple plasmid transfection method was used to produce rAAV vectors as described previously (Dong et al., 2010). Briefly, pAd, pRC and pssAAV-CB-Luciferase or pdsAAV-CB-EGFP were co-transfected into HEK293 cells at a ratio of 2:1:1. The transfected cells were harvested at 72 h post transfection, and the rAAV vectors were purified by two rounds of cesium chloride gradient ultracentrifuge. The purified rAAV vectors were dialyzed in PBS solution containing 5% D-sorbitol for 24 h with two buffer changes, then stored at −80°C before administration. The purity was examined by silver staining and the genome titers were determined by qPCR. The primer pairs targeting the luciferase gene were 5′-TGACCGAGAAGGAGATCGTG-3′and 5′-GAG​AAT​CTC​GCG​GAT​CTT​GC-3’. The primer pairs targeting the EGFP gene were 5′-TGA​CCC​TGA​AGT​TCA​TCT​GC-3′ and 5′-GAA​GTC​GTG​CTG​CTT​CAT​GT-3’.

### Western blot analysis

The primary antibodies used in this study were mouse anti-AAVR (Abcam, ab105385, 1:1,000), and mouse anti-β-actin (Abbkine, ABL1010, 1:2000). The secondary antibody used in this study was HRP conjugated Goat Anti-Mouse IgG (Abbkine, a21010, 1:4,000).

Cell debris were harvested, and cell lysates were prepared on ice using the RIPA lysis buffer (Beyotime, Shanghai, China) supplied with 1% inhibitor cocktail (Bimake, Shanghai, China). The lysates were centrifuged at 10,000 g for 5 min at 4°C and the supernatants were collected. Protein concentrations were quantified by the BCA assay (Thermo Fisher Scientific, Waltham, MA). Twenty μg of total protein was mixed with 6× SDS loading buffer (Sangon, Shanghai, China), followed by heating for 10 min at 95°C. The proteins were separated by SDS-PAGE gel and transferred to a PVDF membrane (Biorad, Irvine, CA). The membrane was blocked in a blocking buffer (PBS with 5% nonfat milk and 0.1% Tween-20) for 1 h at room temperature. Then, the membrane was incubated with a primary antibody overnight at 4°C. The membrane was washed three times with PBST (PBS containing 0.1% Tween-20) and then incubated with an HRP-conjugated secondary antibody for 1 h at room temperature. The membrane was finally washed three times and developed using an ECL kit (Millipore, Temecula, CA).

### RT-qPCR analysis

Total RNAs were extracted from the collected cells using the universal Genomic RNA Extraction Kit (Takara, Dalian, China) and quantified by a Nanodrop. Equal amounts of RNA samples were used for reverse transcription by PrimeScript RT reagent Kit (Takara, Dalian, China). The qPCR reactions were performed using the TB Green Fast qPCR Mix (Takara, Dalian, China). For AAVR amplification we utilized the primer pairs 5′-ACT​CCA​TCA​CCC​TCT​TTG​GG-3′ and 5′-ACT​TTC​CCT​TTG​CTG​CTT​GG-3’. For the internal control GAPDH amplification, the primer pairs were 5′-CAT​CAA​GAA​GGT​GGT​GAA​GCA-3′ and 5′-TCA​AAG​GTG​GAG​GAG​TGG​GT-3’. The AAVR mRNA levels in cells were quantified by the ∆∆Ct method.

### Flow cytometry analysis

Cells were seeded in 24-well plates at a density of 1×10^5^ cells per well and infected with rAAV vectors carrying EGFP. Forty-eight hours after infection, cells were harvested and washed twice with cold PBS. The cells were fixed with 4% PFA for 15 min and centrifuged at 2,000 g for 5 min at room temperature. The cell pellets were suspended in 300 μL of PBS buffer. GFP-positive cells were determined by flow cytometry and the percentage was analyzed by the FlowJo software. The cells without rAAV infection were used as the gating control.

### Measurement of the luciferase activity

Cells were seeded in 96-well plates at a density of 2×10^4^ cells per well and infected by rAAV vectors carrying the luciferase gene. The relative light units (RLU) were detected by the fluorescence microplate reader 48 h post-infection. The cells in 96-well plates were washed with 100 μL of PBS per well and lysed with 100 μL of lysis buffer (Beyotime, Shanghai, China). Then, cell lysates were centrifuged at 10,000 g at 4°C for 5 min, and the supernatants were transferred to a new 96-well plate.

The luciferase activity was measured by a luminescence detector (Biotek, Gaithersburg, MD). Samples were incubated at 37°C with shaking for 15 s to ensure thorough sample mixing. Thirty microliters of D-luciferin substrate (Beyotime, Shanghai, China) was added to each well of a 96-well microplate. The supernatants were transferred to the substrate with an equal volume and mixed gently by pipetting 2–3 times. The luciferase activity was measured using a luminometer after shaking for 15 s, and the RLU were recorded.

### Detection of NAb titers to rAAV

Serum samples were collected from 99 adults. This study was approved by Ethics Committee of West China Hospital (2017, No. 241), Sichuan University, and the informed consents were obtained from patients or their guardians, as appropriate. AAVR-HeLa cells were plated in a 96-well plate at a density of 2.0×10^4^ cells in 100 μL per well, and incubated for 24 h. The serum samples were heated at 55°C for 30 min, and diluted by basic DMEM in a 96-well plate at the ratio of 1:10, 1:30, 1:90, 1:270, 1:810, and 1:2,430 in 120 μL. The rAAV-Luc vectors with different serotypes were diluted to an appreciate titers with PBS in 50 μL. 40 μL of diluted serum was added to 40 μL of rAAV vectors and incubated for one hour in the incubator at 37°C. The basic DMEM was used as the negative control. After incubation, 60 μL of rAAV-serum complex was transferred to the plated cells in triplicate and continued to incubate for 48 h. Finally, the luciferase activity assays were performed. Only the central 60 wells in a 96-well plate were used for titration to avoid the marginal effect.

### Statistical analyses

Two-tailed Student's t-tests were performed using GraphPad Prism and significant levels were defined as *p < 0.05, **p < 0.01, ***p < 0.001.

## Results

### Construction of HeLa stable cells with overexpressed AAVR

**FIGURE 1:**
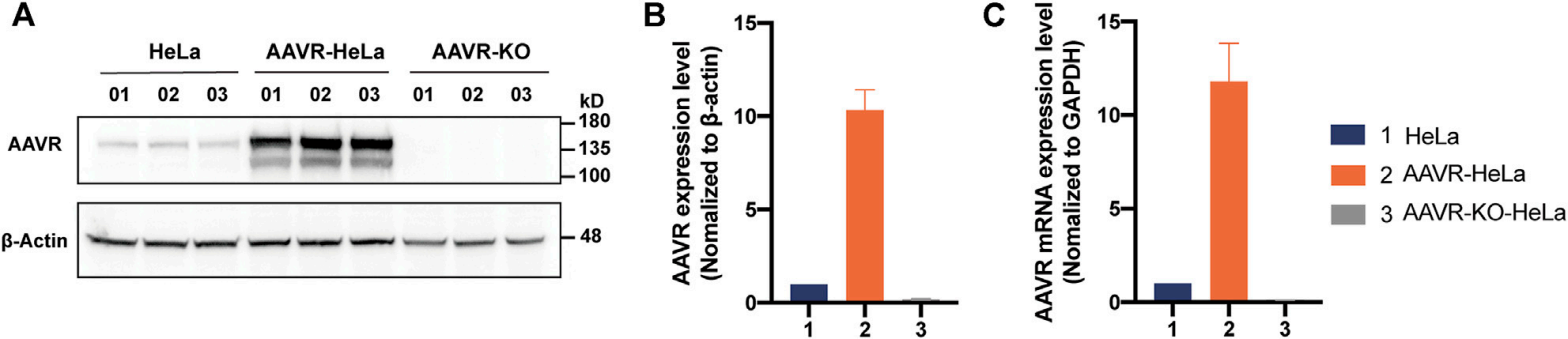
Construction of HeLa stable cells with overexpressed or knockout AAVR. The stable cells with overexpressed AAVR were constructed by infection with a lentiviral vector carrying the full-length AAVR gene. As a control, the stable cells with knockout AAVR were also constructed using the CRISPR/Cas9 gene editing technology. The cells were seeded in triplicate onto six-well plates for protein and RNA extractions. **(A)** A representative Western blotting analysis of AAVR expression levels in HeLa, AAVR-HeLa and AAVR-KO-HeLa cells. **(B)** Fold change of AAVR protein expression levels in the three cell lines. **(C)** Fold change of mRNA expression levels in the three cell lines. RT–qPCR was used for quantification and the expression level of GAPDH was used for normalization (*n* = 3).

As a universal receptor, AAVR mediates the entry of rAAV during transduction, therefore, AAVR overexpressing was hypothesized to be able to improve AAV transduction efficiency for most serotypes. To this end, stable cells with overexpressed AAVR, AAVR-HeLa, were constructed. Meanwhile, the stable cells with knockout AAVR, AAVR-KO-HeLa, were also constructed as a control. Western blotting analysis showed that the expression level of AAVR in AAVR-HeLa cells was ∼10 times higher than that in the parental cells and the expression of AAVR in AAVR-KO-HeLa cells was not detectable (Figures 1A, B). The molecular weight of AAVR was ∼150 kDa which was consistent with the previous study (Pillay et al., 2016). Also, the mRNA expression levels of AAVR were measured by RT-qPCR, and it showed that the AAVR expression level increased 1,100% in AAVR-HeLa cells while it was reduced more than 90% in AAVR-KO-HeLa cells (Figure 1C).

### Enhancement of rAAV transduction efficiency in AAVR-HeLa cells

**FIGURE 2:**
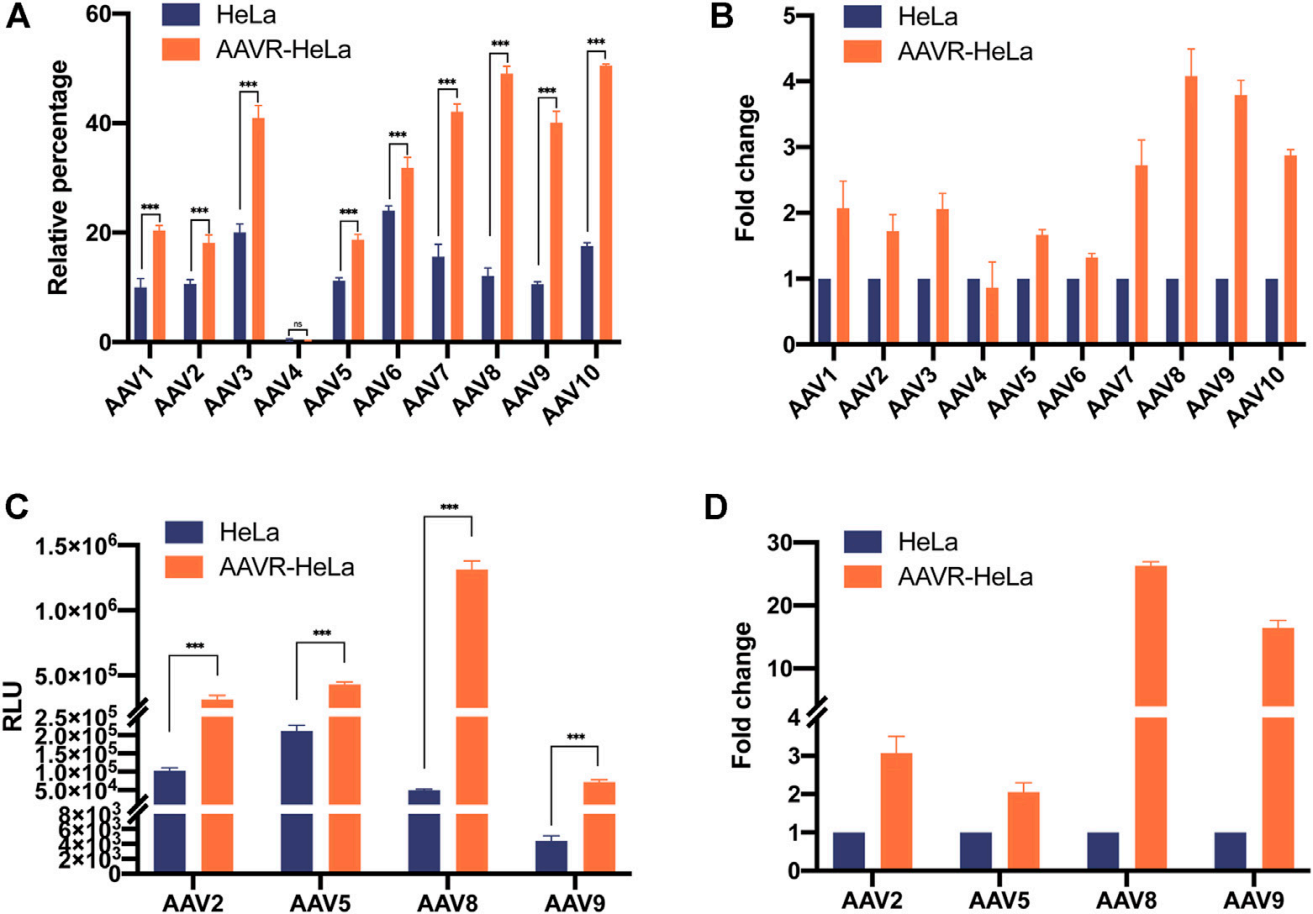
Enhanced transduction efficiency for rAAV-EGFP and rAAV-Luc vectors with different serotypes in AAVR-HeLa cells. The rAAV vectors with different serotypes carrying EGFP or luciferase were used to infect AAVR-HeLa cells. The results were presented as mean ± SD (*n* = 3) and analyzed using GraphPad Prism version 8.4.0 (*p < 0.05, **p < 0.01, ***p < 0.001). **(A)** Transduction efficiencies of rAAV vectors (AAV1-10) carrying EGFP in HeLa and AAVR-HeLa cells. **(B)** The fold changes of rAAV vectors (rAAV1-10) carrying EGFP in AAVR-HeLa cells compared to HeLa cells. **(C)** Transduction efficiencies of the rAAV-Luc in the serotypes of AAV2, AAV5, AAV8, and AAV9 in HeLa and AAVR-HeLa cells. **(D)** The fold changes of rAAV-Luc in the serotypes of AAV2, AAV5, AAV8, and AAV9 in AAVR-HeLa cells compared to HeLa cells.

To characterize the enhancement of AAVR overexpression in rAAV transduction, rAAV vectors carrying EGFP with ten different serotypes (AAV1-10) were used to infect HeLa and AAVR-HeLa cells. Flow cytometry results showed that the transduction efficiencies were significantly increased for all AAV serotypes except AAV4 which nearly could not infect HeLa cells (Figure 2A). The transduction efficiencies for rAAV8-EGFP and rAAV9-EGFP were increased the most, ∼4 fold increase (Figure 2B). For rAAV7 and rAAV10, the increase was ∼3 fold; for rAAV1, rAAV2, and rAAV3, ∼2 fold. The lowest infection increment for rAAV5 and rAAV6, was 1.5 and 1.3 fold, respectively.

To quantify the transduction efficiency increase more accurately, luciferase was used as the reporter gene packaged in AAV2, AAV5, AAV8, and AAV9, the most widely used serotypes in clinical trials. The rAAV-Luc results further supported that the overexpression of AAVR in HeLa cells was able to increase the transduction efficiency for these four rAAV vectors (Figure 2C). Moreover, the fold increases were relatively larger for these vectors. The increase of transduction efficiency was ∼3 fold for rAAV2-Luc and ∼2 fold for rAAV5-Luc (Figure 2D). Notably, the transduction efficiencies were promoted up to 26 and 16 fold for rAAV8 and rAAV9 vectors, respectively (Figure 2D). The rAAV transduction efficiency changes in AAVR-KO-HeLa cells were also examined and it was shown that the transduction for all rAAV vectors with different serotypes were almost completely inhibited (Supplementary Figure S1).

### Stability of the overexpressed AAVR in the AAVR-HeLa cell line

**FIGURE 3:**
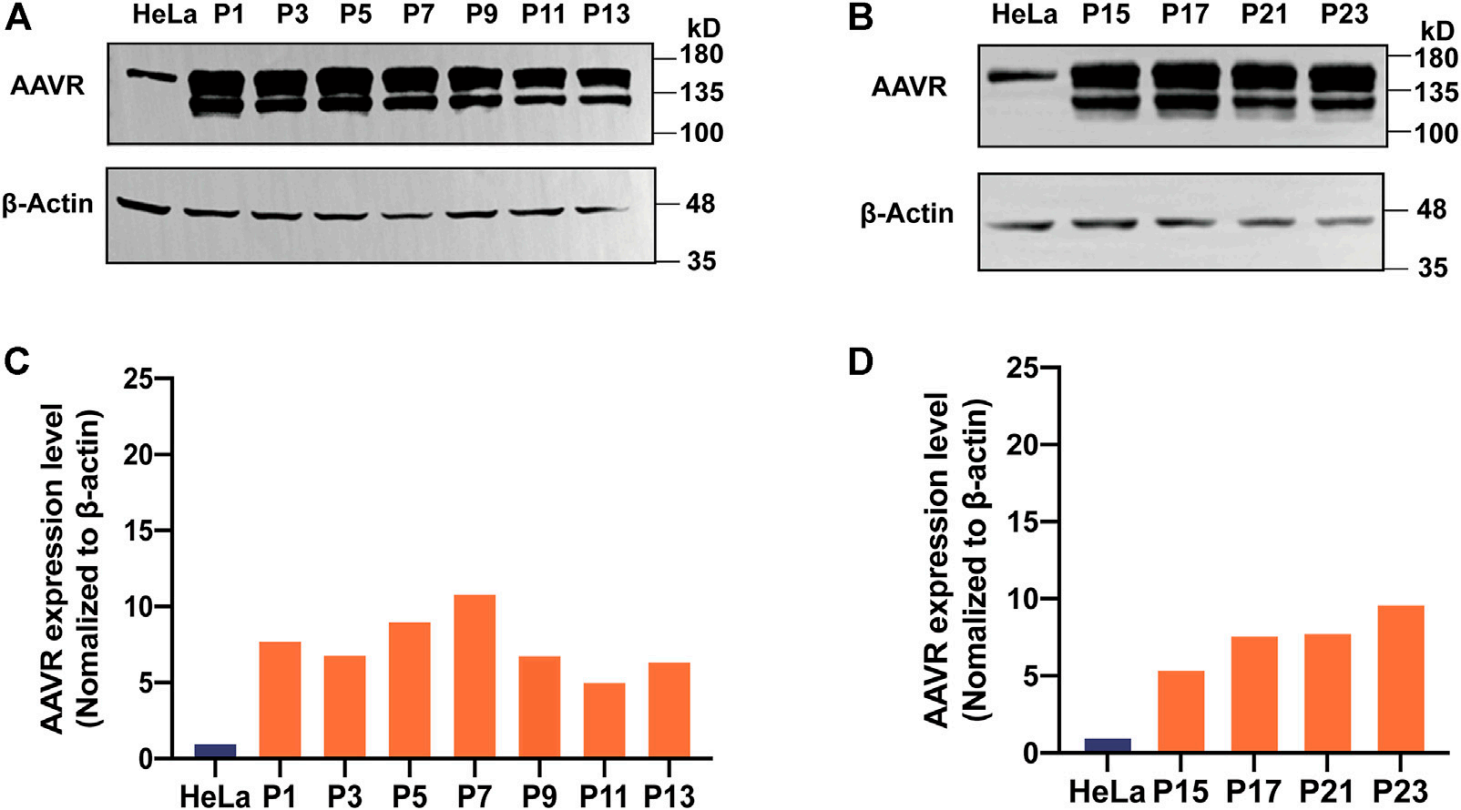
Stability of the overexpressed AAVR in the AAVR-HeLa cell line. The stable cells with overexpressed AAVR were passaged for 23 generations, and the AAVR expression level was analyzed by Western blotting. Cell samples were named according to their passages. **(A)** Western blotting analysis of AAVR expression levels in AAVR-HeLa cells with the passages of P1, P3, P5, P7, P9, P11 and P13. **(B)** Western blotting analysis of AAVR expression levels in AAVR-HeLa cells with the passages of P15, P17, P21, and P23. **(C)** Fold change of AAVR protein expression levels of **(A)**. **(D)** Fold change of AAVR protein expression levels of **(B)**.

Stable performance of a cell line used for AAV NAb detection is crucial. It was observed that the doubling time for the stable cell line, AAVR-HeLa-10, was 24 h which was similar to that of the parental HeLa cells. The amount of AAVR protein in the passage cells with odd numbers were evaluated by Western blot analysis (Figures 3A, B). The gray values of AAVR bands normalized to *β*-actin were calculated by ImageJ (Figures 3C, D). It was shown that AAVR overexpression in AAVR-HeLa-10 cells was stable during the passages.

### Analysis of the transduction specificity of AAVR for lentiviral and adenoviral vectors

**FIGURE 4:**
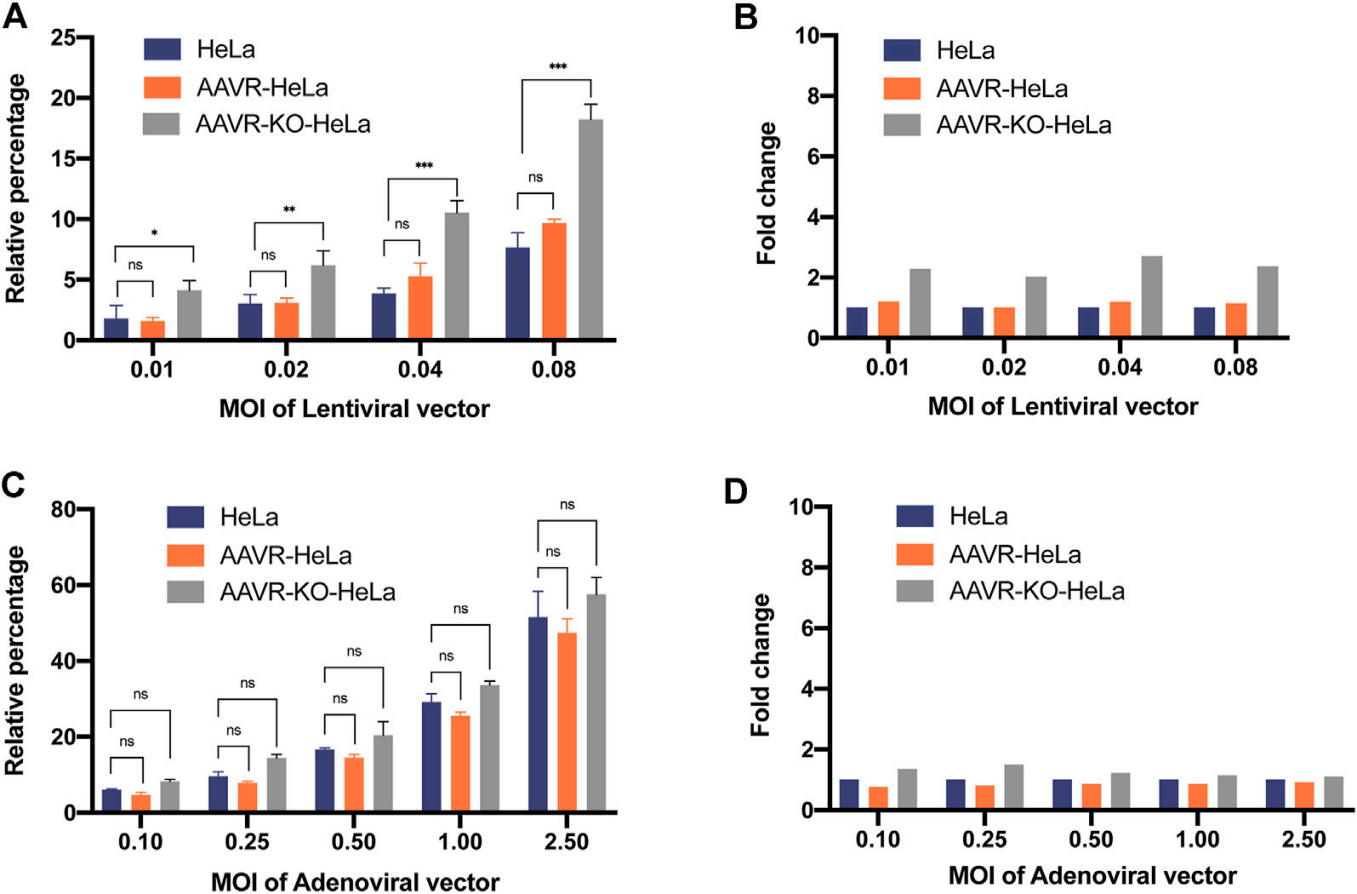
Analysis of the AAVR transduction specificity for lentiviral and adenoviral vectors. The cells were seeded onto 24-well plates and infected by a lentiviral or an adenoviral vector carrying EGFP at the relevant MOIs. EGFP-positive cells were counted by flow cytometry at 48 h post-infection. The results were presented as mean ± SD (*n* = 3) and analyzed by GraphPad Prism version 8.4.0. ns, not significant; *p < 0.05; **p < 0.01; ***p < 0.001. **(A)** Transduction efficiencies of the lentiviral vector carrying EGFP in HeLa, AAVR-HeLa and AAVR-KO-HeLa cells. **(B)** Fold changes of the lentiviral vector carrying EGFP in AAVR-HeLa and AAVR-KO-HeLa cells compared to HeLa cells. **(C)** Transduction efficiencies of the adenoviral vector carrying EGFP in HeLa, AAVR-HeLa and AAVR-KO-HeLa cells. **(D)** Fold changes of the adenoviral vector carrying EGFP in AAVR-HeLa and AAVR-KO-HeLa cells compared to HeLa cells.

To examine whether the overexpression of AAVR enhances the transduction efficiency of other viral vectors, an adenoviral vector and a lentiviral vector, both carrying EGFP reporter gene, were used to infect AAVR-HeLa and HeLa cells. It was demonstrated that the transduction efficiencies for both lentiviral and adenoviral vectors increased and it correlated with the amounts of vector used in the HeLa and AAVR-HeLa cells (Figures 4A, C). However, there were no significant transduction efficiency differences between the lentiviral and adenoviral vectors for HeLa and AAVR-HeLa cells (Figure 4). It suggested that AAVR was not the special receptor for lentiviral and adenoviral vectors. This conclusion was further supported by the results from the adenoviral and lentiviral vectors transductions in AAVR-KO-HeLa and HeLa cells. It was shown that there were no significant differences for the transduction efficiency of the adenoviral vector in the AAVR-KO-HeLa cells compared to the HeLa cells (Figures 4C, D), which was consistent with the previous report (Pillay et al., 2016). Surprisingly, the transduction efficiency was increased for the lentiviral vector in the AAVR-KO-HeLa cells compared to the HeLa cells.

### Enhanced sensitivity of the AAVR-HeLa cell line for AAV NAb detection

**FIGURE 5:**
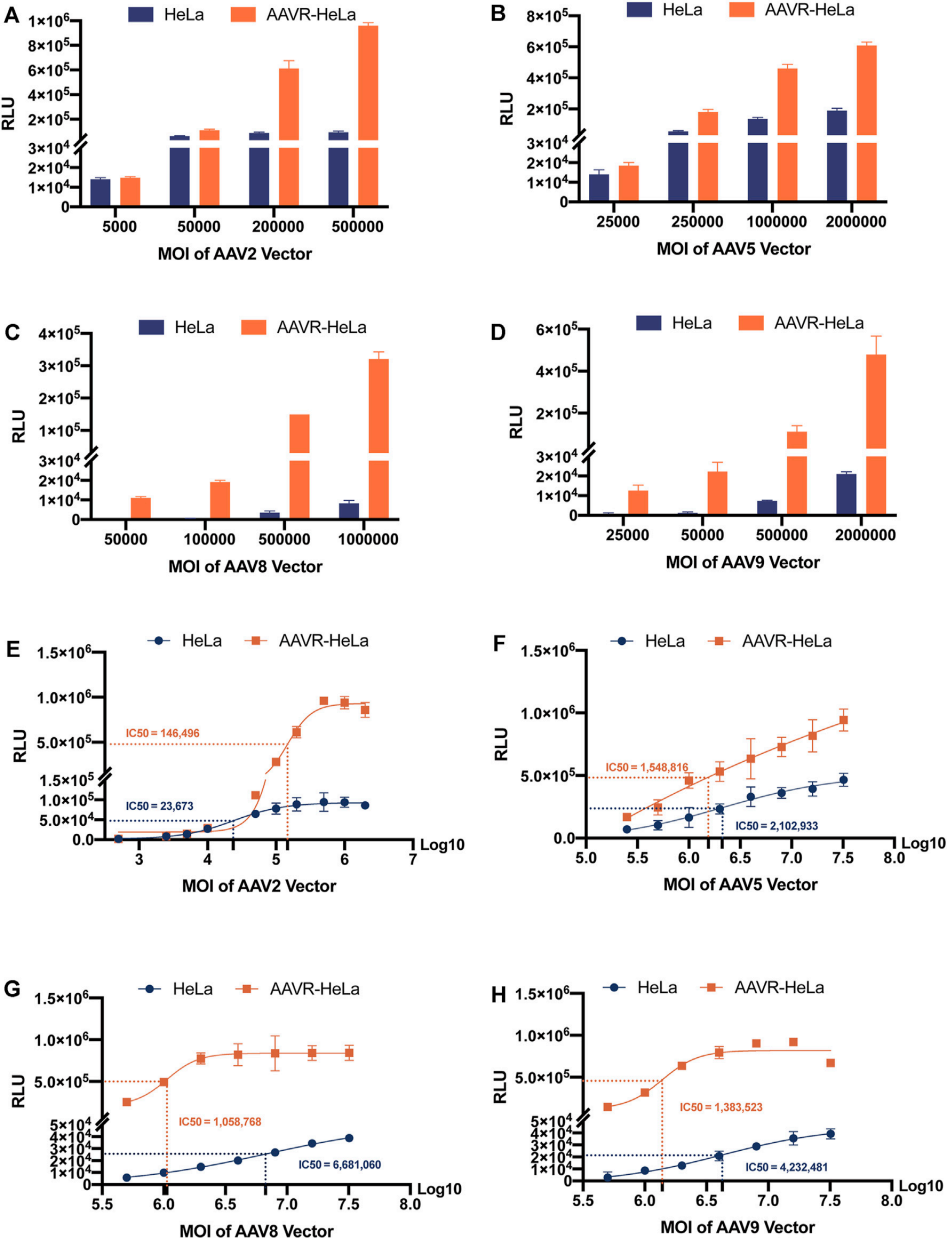
RLU measurements from HeLa and AAVR-HeLa cells infected by rAAV-Luc vectors with different serotypes at variable MOIs. Cells were seeded and infected by rAAV-Luc vectors at the indicated MOIs. Cells were collected at 48 h post-infection and RLUs were measured. For RLU values that did not reach a plateau using MOIs as high as possible under the conditions in this study, the IC_50_ values were given as the AAV MOIs reaching half of the highest RLU values. The results were presented as mean ± SD (*n* = 3). RLU measurements at different MOIs using rAAV-Luc vectors with different serotypes including AAV2 **(A)**, AAV5 **(B)**, AAV8 **(C)**, and AAV9 **(D)**. RLU measurements for IC_50_ calculations using rAAV-Luc vectors with different serotypes including AAV2 **(E)**, AAV5 **(F)**, AAV8 **(G)**, and AAV9 **(H)**.

The NAb titers for the AAV serotype is one of the most essential criteria to exclude patients in gene therapy. A more sensitive NAb detection assay using AAVR-HeLa cells was proposed to be developed at lower MOIs for most serotypes, especially for AAV8 and AAV9. To examine NAb titers, a minimal MOI of rAAV-Luc was considered as the threshold at which 10,000 RLU may be obtained in selected cells (Baatartsogt et al., 2021). To find the appropriate AAV MOIs for NAb detection, the luciferase expression levels in the AAVR-HeLa and HeLa cells were analyzed (Figures 5A–D). In Hela cells, both AAV2 and AAV5 fit the minimal requirement for NAb detection at MOIs of 5,000 and 25,000 (Figures 5A, B), respectively, while AAV8 and AAV9 did not at MOIs of 500,000 (Figures 5C, D). However, in AAVR-HeLa cells, the minimal MOIs for AAV8 and AAV9 were 50,000 and 25,000, which was at least a 10 and 20 fold improvement, respectively (Figures 5C,D). Of note, the transduction saturation lines for AAV2, 5, 8 and 9 were different, which were 500,000, over 32,000,000, 5,000,000, and 5,000,000, respectively.

The IC_50_ was also used to evaluate the sensitivity by Sigmoidal Interpolation analysis (Figure 5). Of note, for RLU values that could not reach the plateau using MOIs that were as high as possible, the IC_50_ values were given as the AAV MOIs reaching half of the highest RLU values measured in this study. It was shown that, for AAV2 that can efficiently transduce HeLa cells, the IC_50_ values did not decrease in AAVR-HeLa cells compared to those in HeLa cells, suggesting that the NAb detection sensitivity for this serotype was not further improved (Figure 5E). For AAV5, the sensitivity improved moderately with an IC_50_ reduction of 26.35% in AAVR-HeLa cells compared to HeLa cells (Figure 5F). However, the sensitivities increased significantly for AAV8 and AAV9, and the IC_50_ values decreased by 84.15%, and 67.31%, respectively (Figures 5G,H).

### Seroprevalence of NAbs to AAV in adults

**FIGURE 6:**
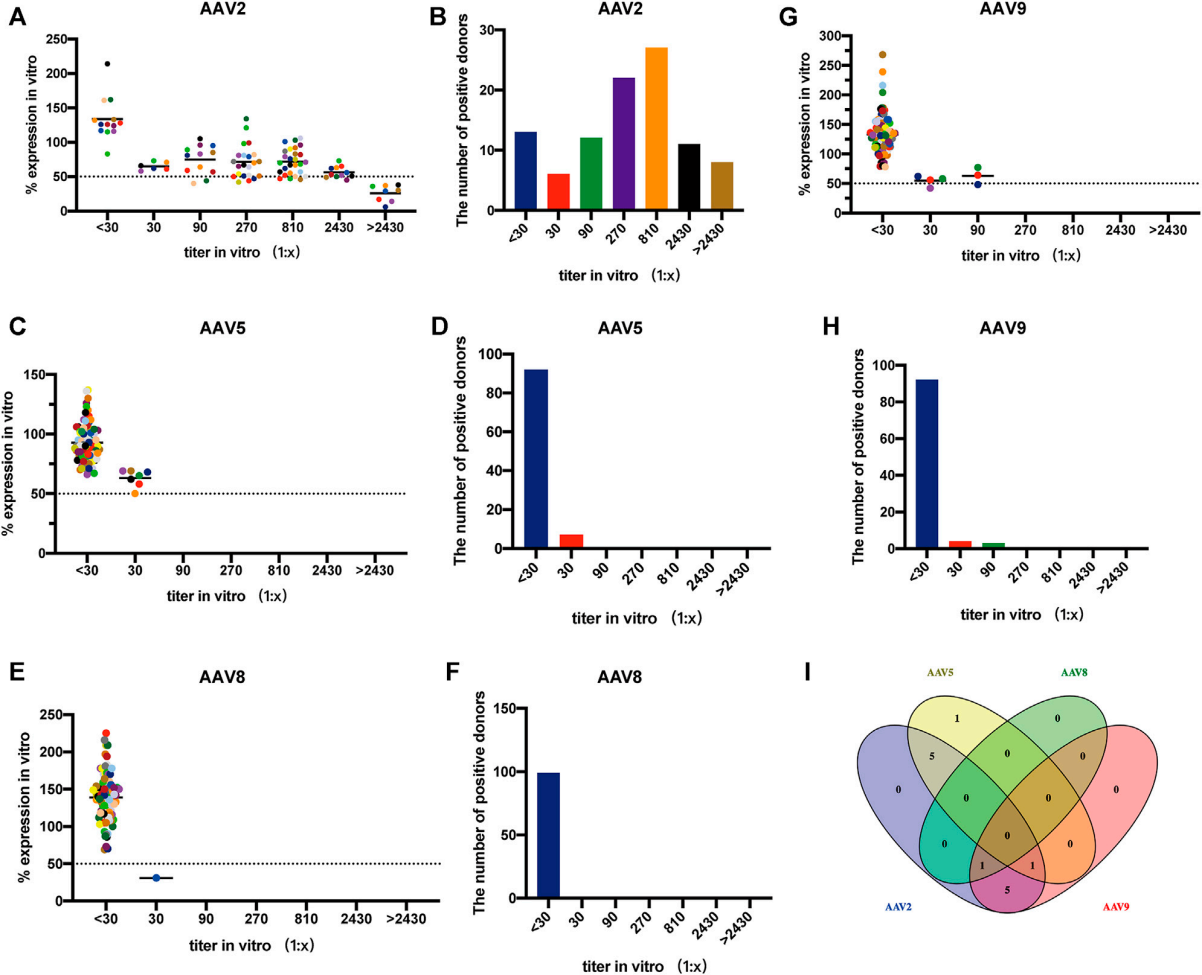
Seroprevalence of NAbs to AAV in adults using AAVR-HeLa cells. The serum samples of 99 Chinese adults were used for the NAb survey. The AAVR-HeLa cells were seeded onto 96-well plates and serial dilutions of serum samples were mixed with rAAV-Luc along with different serotypes. The AAVR-HeLa cells were then infected with the rAAV-serum mix and RLU were measured by the fluorescence microplate reader. The RLU value obtained from the negative control, namely DMEM only, was defined as 100%. RLU inhibition was defined as the percentage of RLU obtained to that from the negative control. ND_50_ values were calculated as the dilutions which inhibited the transduction by 50%. The ND_50_ of NAb titers against AAV2 **(A)**, AAV5 **(C)**, AAV8 **(E)** and AAV9 **(G)** were evaluated, and the corresponding bar charts were also presented **(B, D, F, H)**. **(I)** Venn diagram analysis of the NAb cross-reactivity to different AAV serotypes.

The seroprevalence of NAbs in 99 Chinese adults were surveyed for AAV2, AAV5, AAV8, and AAV9 using AAVR-HeLa cells (Figure 6). It was found that AAV2 was the most prevalent serotype. A total of 86 samples had NAbs against AAV2 (above 1:30) and the seropositive rate was 87%. Most samples had a NAb titer of 1:810, followed by 1:270, which accounted for 27% and 22% of all samples, respectively. Furthermore, the NAb values against AAV2 were over the upper limit in 8 samples (Figures 6A, B). The NAbs to the other three serotypes were lower compared to that to rAAV2. As for AAV5, 92 samples (93%) had NAb titers less than 1:30 (Figures 6C, D). In the case of AAV8, 98 samples (99%) had NAb titers less than 1:30 (Figures 6E, F). For AAV9, 92 samples (93%) had NAb titers less than 1:30 (Figures 6G, H).

Venn diagram analysis was performed to identify the cross-reactivity of NAbs for different AAV serotypes. The NAbs in AAV2-positive samples reacted with the other serotypes, and the number of cross-reactivity for AAV5, AAV8 and AAV9 were 6, 1, and 7, which was accounted for 50%, 8.3% and 58.3% of the 12 samples, respectively. In the 7 samples containing AAV5-positive Nabs, there were 5 and 1 samples that reacted to AAV2 and AAV9, respectively. The NAbs against rAAV8 found in one sample also reacted to both rAAV2 and rAAV9. The cross-reactivity of NAbs to two or three serotypes was observed in 13 samples (13.1%) and none of the sample was found to have NAbs against the four serotypes tested (Figure 6I).

## Discussion

In gene therapy, the NAb titer for AAV serotypes is one of the most important criteria to exclude patients for effective treatment. Herein, we reported the construction of a cell line with overexpressed AAVR, AAVR-HeLa, that improved the detection sensitivity of NAbs. After that, the AAVR-HeLa cell line was utilized to investigate the seroprevalence in Chinese adults.

The NAb assay based on cell-based TI is very popular due to its excellent reproducibility and easiness to perform. However, the infection capacity varies largely for different rAAV serotypes, and may be under the limit of detection for several serotypes including rAAV8 and rAAV9. To address this challenge, different cell lines were chosen to increase the assay sensitivity, including HEK293, COS-7, GM16095 and HeLa cells, in which HeLa cells are widely used for many serotypes (Kay et al., 2000; Meliani et al., 2015; Guo et al., 2019; Daniel et al., 2022). In this study, a new approach to increase the sensitivity of cell-based TI assay was demonstrated by using AAVR-HeLa cells in which AAVR was overexpressed. Compared to HeLa cells, the transduction efficiencies in AAVR-HeLa increased 26 and 16 fold for rAAV8 and rAAV9, respectively, increasing the sensitivity of TI assays for these two serotypes that are difficult to transduce. For highly transducible rAAV2 and rAAV5 in AAVR-HeLa, the infection efficiencies further increased two to three fold. In NAb assay, higher transduction efficiency means lower amount of viral vector could be used, so the sensitivity of the assay increases (Figures 5E–H). Significantly, the AAVR gene was demonstrated to be stable in HeLa after at least 23 passages. The AAVR-HeLa stable cell line constructed in this study could be used for NAb detection for most of AAV serotypes, which made this assay easier and more cost effective.

Different reporter genes are packaged in rAAV vectors and used in the cell-based TI including EGFP, LacZ and luciferase (Mingozzi et al., 2013; Falese et al., 2017; Wang et al., 2018; Weber, 2021). Among them, luciferase is the most popular because its quantification is simple and more accurate, as was demonstrated in this study (Figure 2). Nano-Luc, a report gene which is more sensitive than luciferase, was applied recently used to detect NAbs by cell-based TI (Baatartsogt et al., 2021). Thus, the sensitivity of NAb detection may be further improved using a Nano-Luc and AAVR-HeLa cell combination. After characterization of AAVR-HeLa cells, NAbs against AAV2, AAV5, AAV8 and AAV9, the four most used serotypes utilized in gene therapy, were investigated (Figure 6). NAbs against AAV2 were observed in a higher proportion of human participants than those against to the other three serotypes, which is consistent with many other studies (Calcedo et al., 2009; Mingozzi et al., 2013; Wang et al., 2018). A study has shown that the highest NAbs prevalence in the selected cohort was AAV2 (59%), followed by AAV1 (50.5%) (Boutin et al., 2010). The lowest NAb titers were observed for AAV8 (19%) and AAV5 (3.2%) (Boutin et al., 2010). Although our seroprevalence values were different compared to other studies, it may partly be explained by the cohort selection or sample size. Remarkably, no serum sample contained NAbs for all four serotypes tested in this study. This indicates that even the patient was positive for some AAV serotypes, there may one or more serotypes that could be available for gene therapy for a number of individuals.

One limitation of this study was the lack of data relevant to a serum dilution ratio of 1:5 which is the standard criteria in clinical trials. One reason was that the rAAV transduction efficiency was enhanced by < 1:30 diluted serum under our NAb evaluation condition. It could be that the negative control was diluted by DMEM media but not by negative sera. Negative sera contain the similar components as the tested serum, which may be a better matrix for dilution. Another limitation was that the MOIs of rAAV-Luc used for NAb detection were not optimized to identify a suitable, lower dosage for each serotype. A study found that, at the same dilution ratio, ND_50_ increased more at MOI of 100 than at MOI of 1,000 (Baatartsogt et al., 2021). Thus, it is desired that the amount of the viral vectors is used as little as possible for more accurate NAb assay. The third limitation of this study was that the IC_50_ values for AAV5, 8 and 9 were not calculated precisely because the RLU plateaus did not appear even 32 μL of the viral vectors with the titer of 2×10^13^ VG/mL were used. It was close to the highest amount of a viral vector could be added in a well of the 96-well plate.

In conclusion, the AAVR-HeLa cell line was constructed and AAVR could be stably overexpressed for 23 passages. This cell line was able to increase the detection sensitivity of NAbs to most AAV serotypes, which is especially useful for AAV8 and AAV9 that are difficult to transduce cells.

## Data Availability

The original contributions presented in the study are included in the article/Supplementary Material, further inquiries can be directed to the corresponding authors.
