# Supplementary material for: Enhanced sensitivity of neutralizing antibody detection for different AAV serotypes using HeLa cells with overexpressed AAVR
Source: Front Pharmacol. 2023 Apr 27;14:1188290. doi: 10.3389/fphar.2023.1188290 (PMC10176094; doi:10.3389/fphar.2023.1188290)
Supplement: Supplementary file 1 [file Table1.DOCX]

Supplementary Material

Enhanced Sensitivity of Neutralizing Antibody Detection for Different AAV Serotypes using HeLa Cells with Overexpressed AAVR

**Zhaoyue Zheng****^1†^, Jingya Ye^1†^, Mi Leng^1^, Chunmei Gan^1^, Na Tang^2^, Wei Li^3^, C. Alexander Valencia^1^, Biao Dong^1,2*^and Hoi Yee Chow^1*^**

^1^National Clinical Research Center for Geriatrics and State Key Laboratory of Biotherapy, West China Hospital, Sichuan University, Chengdu, China

^2^Sichuan Real & Best Biotech Co., Ltd, Chengdu, China

^3^Department of Dermatovenereology, Rare Disease Center, West China Hospital, Sichuan University, Sichuan, China

***Correspondence:**Hoi Yee Chow

[ahyeechy@gmail.com](mailto:ahyeechy@gmail.com)

Biao Dong

[biaodong@scu.edu.cn](mailto:biaodong@scu.edu.cn)

†These authors contributed equally to this work and share first authorship

## Supplementary Figures


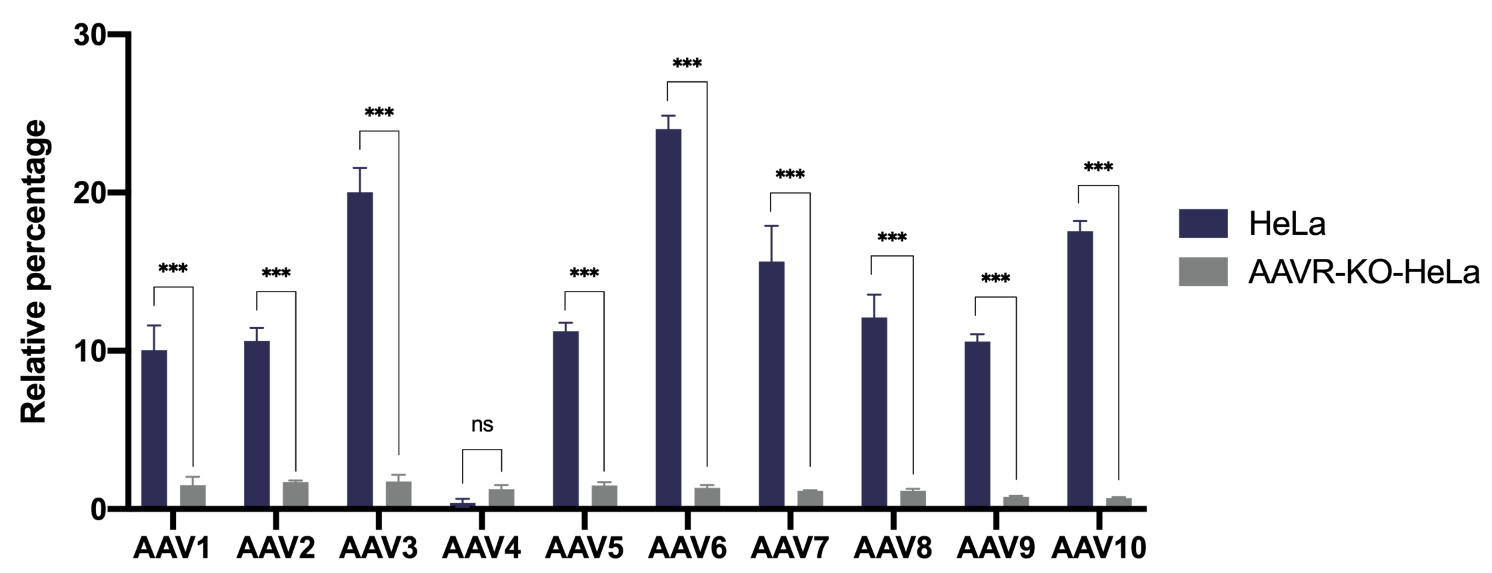


**Supplementary Figure 1.** **Inhibited transduction efficiency for rAAV-EGFP vectors with different serotypes in AAVR-KO-HeLa cells.** The rAAV vectors with different serotypes carrying EGFP were used to infect AAVR-KO-HeLa cells and HeLa cells. The results were presented as mean ± SD (n = 3) and analyzed using GraphPad Prism version 8.4.0 (P<0.001).
